# Ultrasound guided double injection of blood into cisterna magna: a rabbit model for treatment of cerebral vasospasm

**DOI:** 10.1186/s12938-016-0123-z

**Published:** 2016-02-06

**Authors:** Yongchao Chen, Youzhi Zhu, Yu Zhang, Zixuan Zhang, Juan Lian, Fucheng Luo, Xuefei Deng, Kelvin KL Wong

**Affiliations:** Ultrasound Center, The 105th Hospital of PLA, Hefei, China; Department of Radiology, The 105th Hospital of PLA, Hefei, China; Department of Anatomy, Anhui Medical University, Hefei, China; School of Medicine, Western Sydney University, Sydney, Australia

**Keywords:** Cerebral vasospasm, Double injection of blood into cisterna magna, Animal model, Ultrasound guided puncture

## Abstract

**Background:**

Double injection of blood into cisterna magna using a rabbit model results in cerebral vasospasm. An unacceptably high mortality rate tends to limit the application of model. Ultrasound guided puncture can provide real-time imaging guidance for operation. The aim of this paper is to establish a safe and effective rabbit model of cerebral vasospasm after subarachnoid hemorrhage with the assistance of ultrasound medical imaging.

**Methods:**

A total of 160 New Zealand white rabbits were randomly divided into four groups of 40 each: (1) manual control group, (2) manual model group, (3) ultrasound guided control group, and (4) ultrasound guided model group. The subarachnoid hemorrhage was intentionally caused by double injection of blood into their cisterna magna. Then, basilar artery diameters were measured using magnetic resonance angiography before modeling and 5 days after modeling.

**Results:**

The depth of needle entering into cisterna magna was determined during the process of ultrasound guided puncture. The mortality rates in manual control group and model group were 15 and 23 %, respectively. No rabbits were sacrificed in those two ultrasound guided groups. We found that the mortality rate in ultrasound guided groups decreased significantly compared to manual groups. Compared with diameters before modeling, the basilar artery diameters after modeling were significantly lower in manual and ultrasound guided model groups. The vasospasm aggravated and the proportion of severe vasospasms was greater in ultrasound guided model group than that of manual group. In manual model group, no vasospasm was found in 8 % of rabbits.

**Conclusions:**

The ultrasound guided double injection of blood into cisterna magna is a safe and effective rabbit model for treatment of cerebral vasospasm.

## Background

Despite the advances in diagnosis and treatment of subarachnoid hemorrhage, effective therapeutic interventions are still limited and clinical outcomes remain disappointing [1]. There is substantial evidence that delayed cerebral vasospasm contributes to the significant mortality and morbidity rates following subarachnoid hemorrhage [1–9]. Cerebral vasospasm can lead to cerebral hypoperfusion, culminating in delayed ischemic neurological deficit, which has been considered a major cause of high mortality and poor outcome. In the last several decades, many researchers have been primarily focused on vasospasm and its sequelae [1–16]. However, the success rate with regard to improved outcome is also limited [1]. For a better understanding of the pathogenic mechanism of cerebral vasospasm and to develop efficacious therapeutic strategies, many animal models have been stimulated [17–20].

Choosing an appropriate animal model is a critical step in the productive cerebral vasospasm research [21]. Primates may be the most preferred species, as the time course of delayed cerebral vasospasm is similar to that observed in humans and the angiography is relatively easy to perform [20]. However, drawbacks with these species include high costs, limited availability, and difficulties in inducing subarachnoid hemorrhage. Canines are another suitable species, especially when used in the molecular biology research [22]. However, the canines are too small and the angiography is hard to perform. Therefore, rabbits are the alternative species of choice, as they offer many advantages [22–26]. First, the time course of cerebral vasospasm shows a biphasic pattern of early and delayed vasospasm as found in humans. Second, the morphological changes in arteries and ventricles observed in rabbit models are similar to those observed in humans. Third, rabbit models are available in larger numbers with relatively low costs, and intubation and respiratory support are not required in anesthesia. Finally, rabbits are relatively easy to restrain when using an appropriate restraining device, given their relatively docile nature.

In the 1980s, rabbits emerged as a new species in delayed cerebral vasospasm research. Since Liszczak et al. presented models of blood injection into cisterna magna [27], this technique became the standard for subarachnoid hemorrhage induction in rabbits. It can generate a pathologic condition similar to that seen after the rupture of an intracranial aneurysm and it is easy to perform with a high success rate. The frequency of blood injection ranges from 1 to 3 times. Double injection method induces a more severe and prolonged vasospasm than single injection method, and is well established in dog and rat models [17, 19, 20, 22]. In rabbits, double injection method reportedly produces a vasospasm that is more severe and persistent. The vasospasm peaks approximately 5 days after first injection and persists for up to the next 2 days [21]. However, due to the unacceptably high mortality rate, reported by Baker et al. (41 %) [28], Spallone and Pastore (20 %) [29], double injection method is not popular in rabbit models.

Deaths of animal occurring during the procedure are mainly caused by a failure to the brainstem needling or intraparenchymal blood injection [26]. Various methods have been used to avoid puncture failure such as having an appropriate posture, not evacuating too much cerebrospinal fluid, directing the needle slightly rostrally [25, 26]. However, deaths of animal were inevitable as the cisterna magna is small and the location of needle tip cannot be confirmed. Moreover, double injection method tends to increase the risk of death [25]. Ultrasound guided puncture is a widely used interventional technique in the clinic, which provides real-time imaging guidance for all kinds of puncture [30–36]. The present study was designed to use ultrasound guided puncture in the establishment of cerebral vasospasm model so as to provide a safe and effective rabbit model of cerebral vasospasm after subarachnoid hemorrhage.

## Methods

### Experimental animal grouping and development of subarachnoid hemorrhage model

This study was approved by the Ethics Committee of Anhui Medical University (Hefei, China, 2012238). All animal use and care protocols including the operation procedures were carried out in strict accordance with the recommendation in Guide for the Care and Use of Laboratory Animals of the National Institutes of Health.

**Fig. 1:**
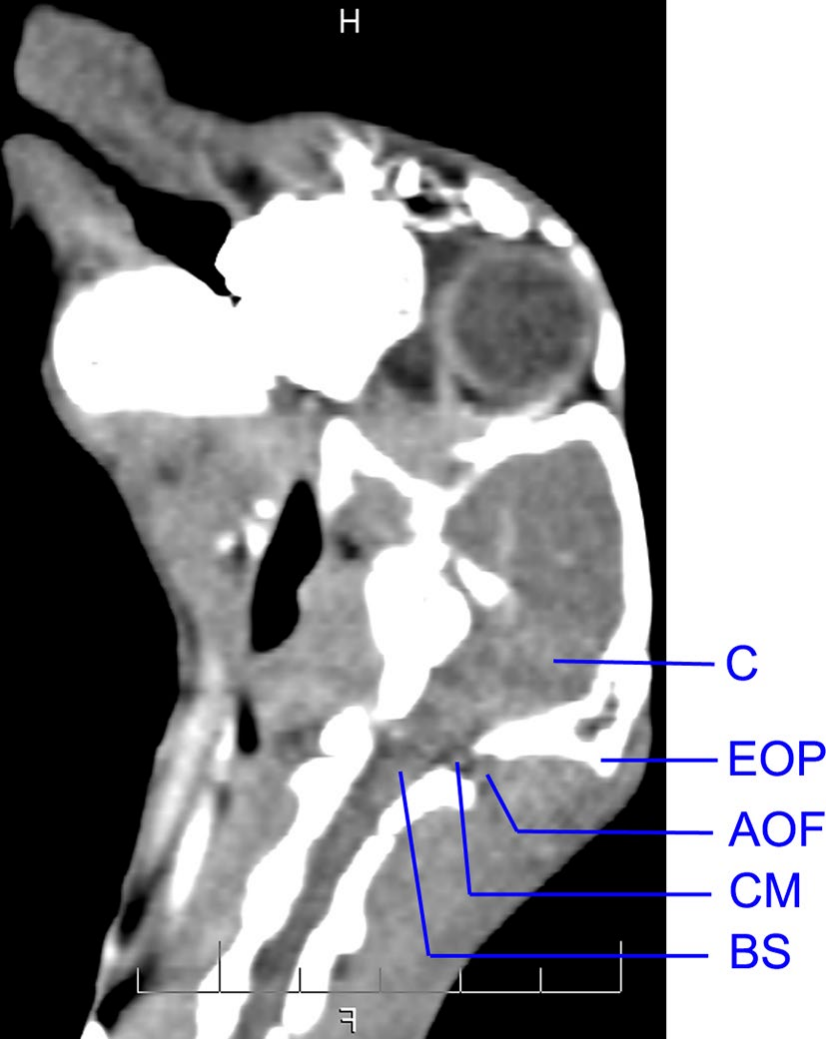
Cisterna magna and atlano-occipital fascia in CT sagittal image. *C* cerebellum, *EOP* external occipital protuberance, *AOF* atlano-occipital fascia, *CM* cisterna magna, *BS* brain stem

A total of 160 adult male New Zealand white rabbits that weigh from 2.5 to 3.2 kg were supplied by the experimental animal center of Anhui Medical University. All rabbits were randomly divided into four groups of 40 each: (1) manual control group, (2) manual model group, (3) ultrasound guided control group, and (4) ultrasound guided model group. We established and detailed the groups as follows:

*Manual model group*. After rabbits were fixed on the operating table, they were anesthetized using 3 % sodium pentobarbital (1 ml/kg) after an intravenous injection through auricular vein. The occipital hair was shaved, and the skin was sterilized with 75 % alcohol. A 5 ml-syringe with a 22-gauge needle was inserted into cisterna magna through atlano-occipital fascia (Fig. 1). Once the dura mater was perforated, a small amount of cerebrospinal fluid (about 0.4 ml/kg) was removed. The autologous non-heparinized fresh auricular arterial blood (about 0.6 ml/kg) was injected into cisterna magna slowly. The rabbits were kept in head-down position for 30 min, whereby the blood would distribute into other subarachnoid spaces with cerebrospinal fluid circulation. The second injection was accomplished 48 h after the first injection, and 0.4 ml/kg blood was injected into cisterna magna using the same procedure as first injection.

*Manualcontrolgroup*. All the procedures were the same as model group, except normal saline was used instead of autologous blood.

**Fig. 2:**
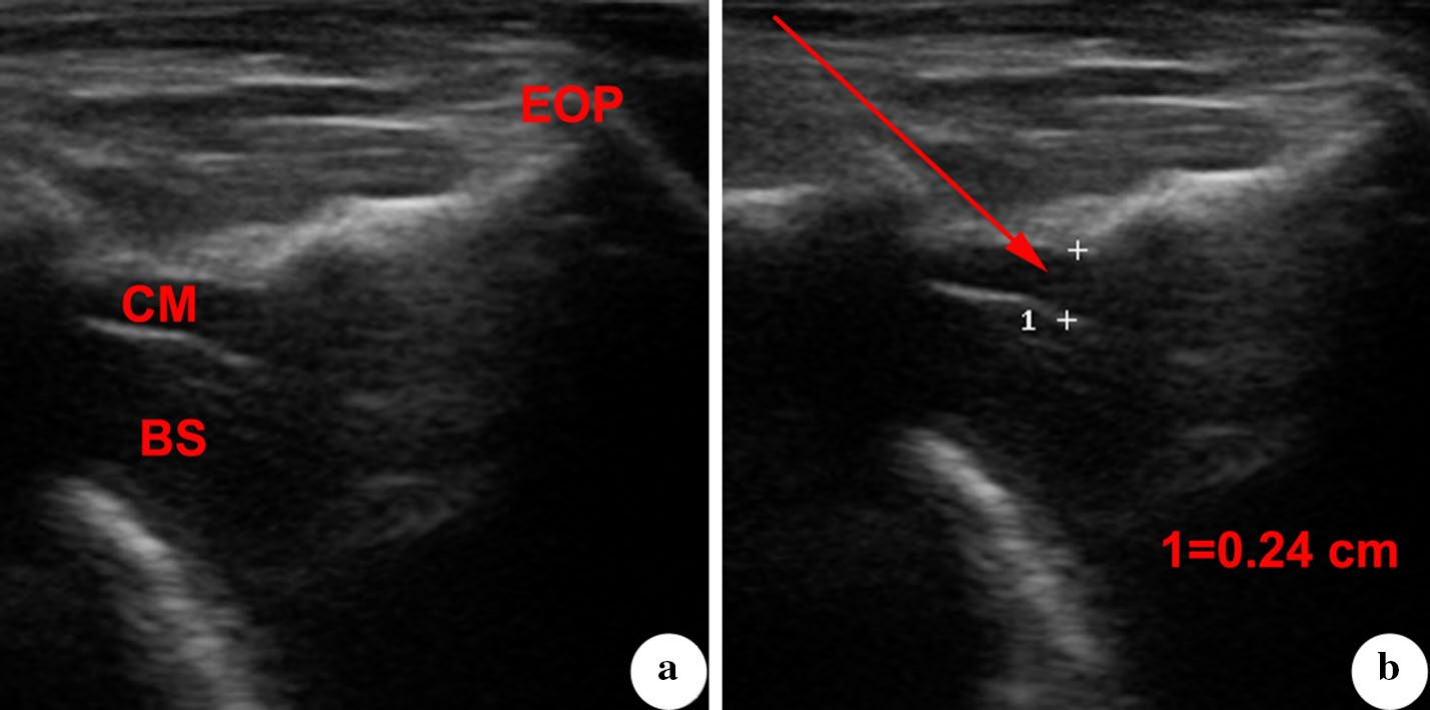
Measurement and observation of cisterna magna that is guided by ultrasound. **a** Cisterna magna and adjacent structures were displayed by ultrasound. **b** Depth of cisterna magna was measured at its optimal part. The puncture direction was designed based on the *line* from atlano-occipital fascia to the deepest part of cisterna magna. *CM* cisterna magna, *BS* brain stem, *EOP* external occipital protuberance

*Ultrasound guided model group*. Before puncture, the depth of cisterna magna was measured by ultrasound. The best puncture direction was designed according to the relationship between atlano-occipital fascia and the deepest part of cisterna magna (Fig. 2). If the cisterna magna was found to be too small (the depth was less than 0.23 cm, which is the length of inclined plane of needle tip), the amount of autologous blood injected into cisterna magna would be decreased. Guided by ultrasound, the needle was inserted into cisterna magna. Other procedures remained the same as manual model group.

*Ultrasound guided control group.* All the procedures were the same as model group except that normal saline was used instead of autologous blood.

### Observation of the rabbit subarachnoid hemorrhage model under CT

The rabbits underwent CT scanning 1 day after modeling, to determine whether there was blood in the subarachnoid space. All CT images were obtained using a 16-MDCT unit (Siemens Healthcare). The scanning parameters were as follows: 80 kV, 100 mA, 1.25-mm section thickness, 1-mm intersection gap, 0.7 cm/s table speed, 9.6-cm FOV, and 512 × 512 matrix.

### Observation of cerebral vasospasm with magnetic resonance angiography

Next, the rabbits underwent MR scanning before modeling and 5 days after modeling, to determine whether cerebral vasospasm occurred. MR scanning was performed with a 3.0 T MR unit (Siemens Healthcare) using a knee joint coil. After a T2-weighted sequence with a TR/TE of 2000/96 ms was performed to display the loose connective tissue, the time of flight magnetic resonance angiography (TOF-MRA) was performed to display cerebral artery. The scanning parameters were as follows: 256 × 256 matrix, 0.5-mm section thickness, 11-cm FOV, TR 25 ms, and TE 5.72 ms. The data were translated to post-processing workstation after scanning. The basilar artery diameters were measured by two radiologists independently, and their mean values were calculated.

The vasospasm severity was calculated based on the basilar artery diameters before modeling and 5 days after modeling. The calculation formula was modified from Laslo et al. [37] in Eq. (1) as follows:

1 $${\text{Vasospasm severity }} = \, \left( {{\text{BA}}_{0} {-}{\text{ BA}}_{ 5} } \right) \, \times { 1}00\% \, /{\text{ BA}}_{0}$$

In this formula, BA_0_ represents the basilar artery diameter before modeling, while BA_5_ represents the basilar artery diameter 5 days after modeling in the same rabbit.

### Statistical analysis

All the statistical analyses were performed with SPSS software for Windows, and are presented as the mean ± standard deviation (SD). Statistical comparisons of the basilar artery diameter before and after modeling were made using paired *t* test. Statistical comparisons of the mortality rate and the vasospasm severity were made using Chi square test. Here, *P* < 0.05 was considered to indicate a significant difference.

## Results

### Operation based on ultrasound guided puncture

**Fig. 3:**
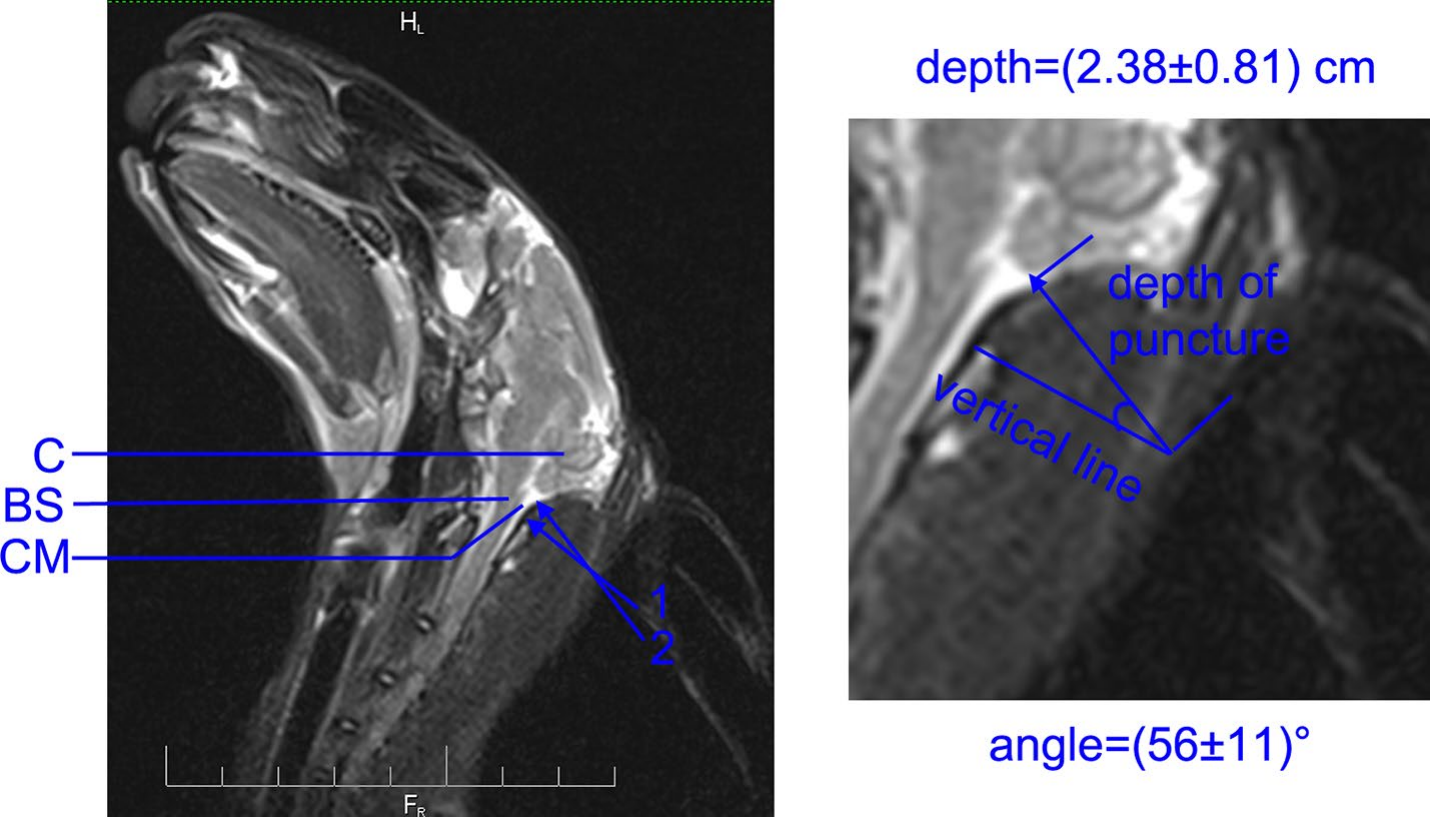
Illustration of puncture direction in MR sagittal image. *C* cerebellum, *CM* cisterna magna, *BS* brain stem, *Arrow 1* puncture direction in manual model group (*vertical line*), *Arrow 2* puncture direction in ultrasound guided model group

The cisterna magna was a dilated subarachnoid space between cerebellum and medulla oblongata (Fig. 3). Before the puncture procedure, the cisterna magna, brain stem and external occipital protuberance were displayed by ultrasound. The depth of the cisterna magna was (0.42 ± 0.06) cm (Fig. 2). According the line from atlano-occipital fascia to the deepest part of cisterna magna, the best puncture direction was designed. The angle between puncture needle and vertical line was (56 ± 11)°, while the depth of puncture was (2.38 ± 0.81) cm (Fig. 3).

**Fig. 4:**
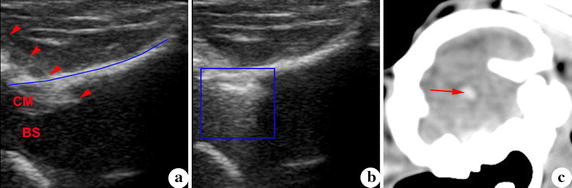
Ultrasound guided puncture procedure. **a** Needle was showed in the puncture procedure. **b** When blood was injected, the signal of flow blood was monitored by ultrasound, and boundary between cisterna magna and brain stem was disturbed by the high signal. **c** Blood in subarachnoid space at 1 day after modeling. *CM* cisterna magna, *BS* brain stem, *Blue line* dura mater, *Red arrowhead* puncture needle, *Blue block* blood signal, *Red arrow* blood in subarachnoid space

During ultrasound guided puncture procedure, the whole process of the needle entering into cisterna magna was observed directly. The depth of needle tip entering into cisterna magna and the distance between tip and brain stem were controlled by operator (Fig. 4a). When blood was injected into cisterna magna, the signal of flow blood was monitored by ultrasound (Fig. 4b). At 1 day after modeling, the blood was observed in subarachnoid space in CT images (Fig. 4c), which illustrated that the injection was successful.

### Safety considerations of puncture

For the manual control group (40 rabbits), six rabbits died within 30 min after puncture. Two died in the first puncture, while the other four died during the second puncture. The mortality rate in manual control group was 15 %. For the manual model group (40 rabbits), nine rabbits died within 30 min after puncture, while six rabbits died during the second puncture. The mortality rate in manual model group was 23 %. There was no statistical difference between the mortality rate of manual control group and manual model group (x^2^ = 0.738, *P* = 0.390).

**Fig. 5:**
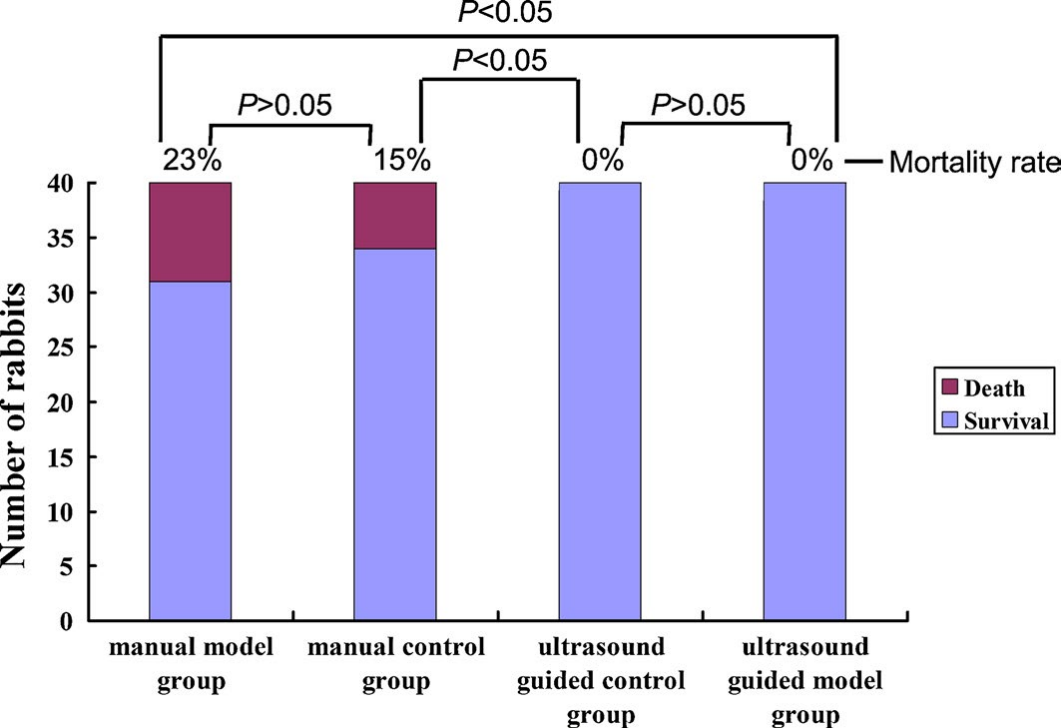
Comparison of mortality rates among four groups

There were no deaths among all 80 rabbits in ultrasound guided control group and model group. Compared with manual model group, the mortality rate in ultrasound guided model group was significantly lower (x^2^ = 10.141. *P* = 0.002). The same difference was observed between manual control group and ultrasound guided control group (x^2^ = 6.486. *P* = 0.026). Comparison of the mortality rates between all four groups was shown in Fig. 5.

### Effectiveness of rabbit cerebral vasospasm model

**Fig. 6:**
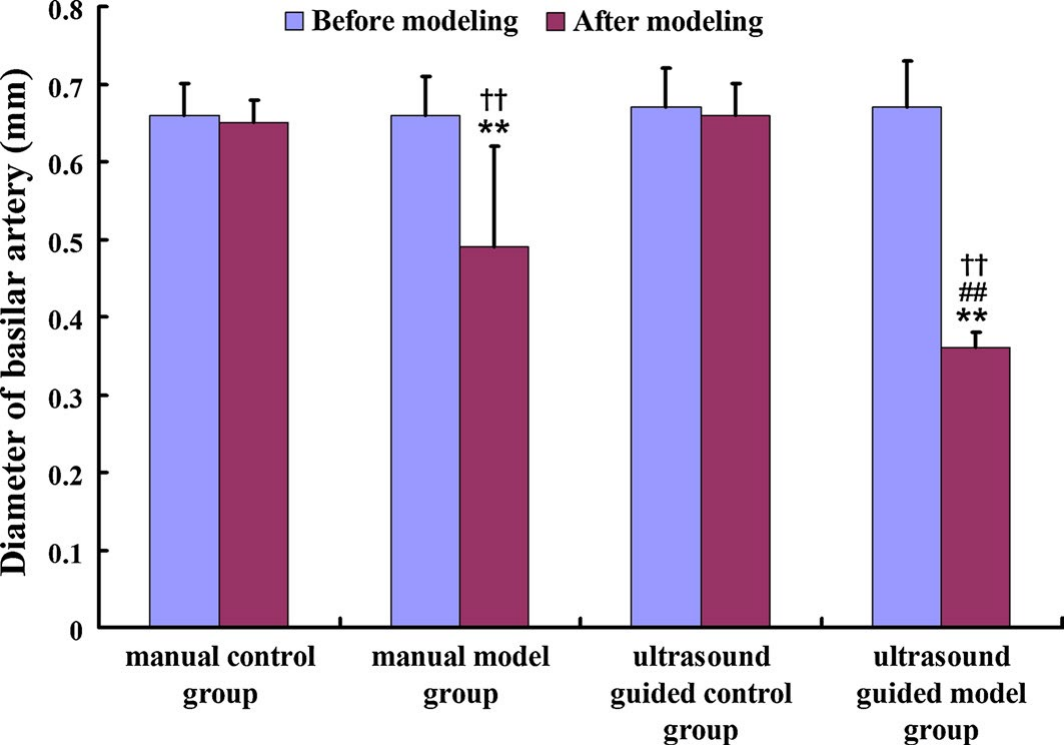
Comparison of basilar artery diameter among four groups. **Compare with the diameter before modeling, *P* < 0.05. ^##^Compare with the diameter in the manual group, *P* < 0.05. ^††^Compare with the diameter in the control group, *P* < 0.05

**Fig. 7:**
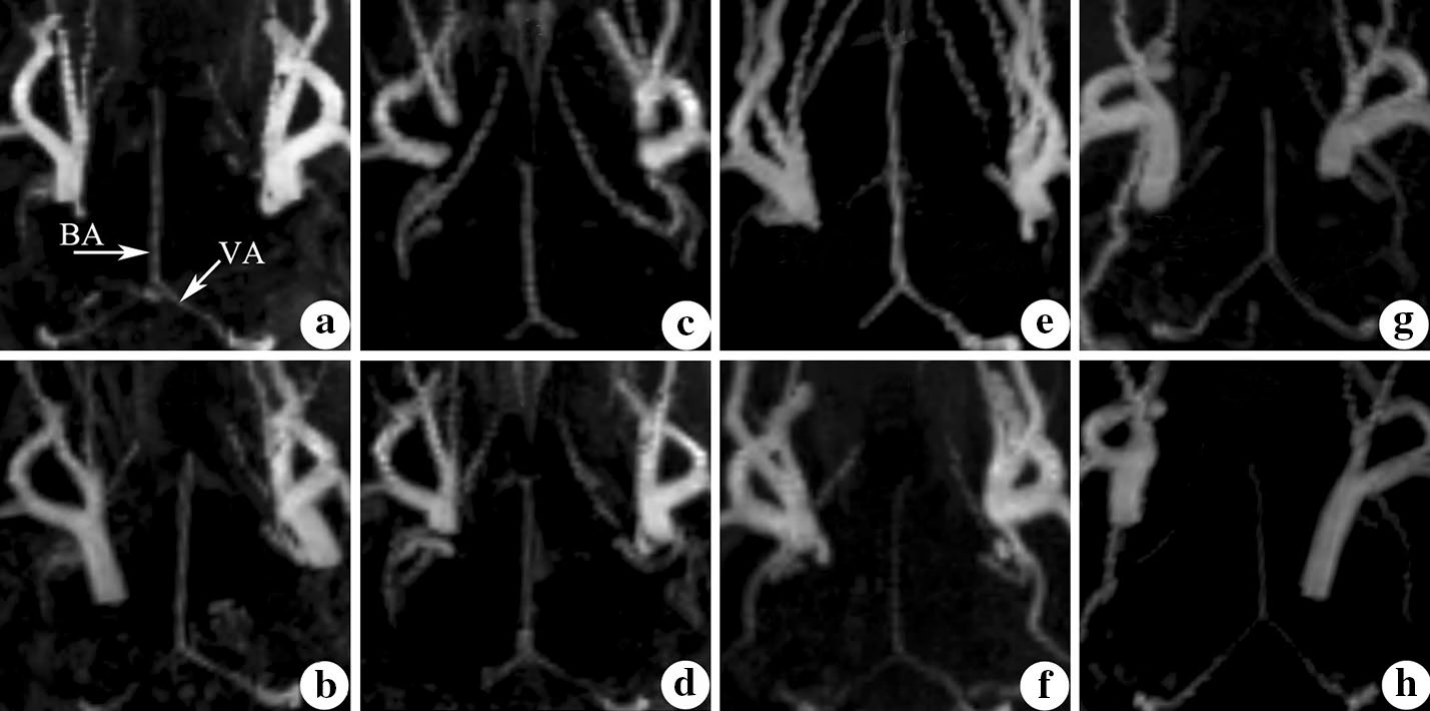
Observation of basilar artery in MRA before and after modeling. **a, b** Manual control group. **c, d** Ultrasound guided control group. **e, f** Manual model group. **g, h** ultrasound guided model group. **a, c, e, g** Before modeling. **b, d, f, h** 5 days after modeling. *BA* basilar artery, *VA* vertebral artery

In both the manual and ultrasound guided control groups, there were no statistical differences in basilar artery diameter between the day before and after modeling. Before modeling, the basilar artery diameter in manual model group was (0.66 ± 0.05) mm, while the diameter was (0.67 ± 0.06) mm in ultrasound guided model group. Five days after modeling, the diameter changed to (0.49 ± 0.13) and (0.36 ± 0.02) mm in manual model group and ultrasound guided model group, respectively. Compared with the diameter before modeling, the basilar artery was significantly narrower after modeling, in both the manual and ultrasound guided model groups. However, the change in ultrasound model group was larger than that in manual guided model group (Figs. 6 and 7).

After calculating the vasospasm severity, the mean severity in manual model group and ultrasound guided model group was 26 and 46 %, respectively. The proportions of the severe (≥30 %), moderate (20 ~ 29 %), mild (10 ~ 19 %) and little (<10 %) were shown in Table 1. The proportion of severe vasospasms was more in ultrasound guided group than that of manual group (x^2^ = 12.917, *P* = 0.001). In the ultrasound guided group, the depth of cisterna magna in two rabbits was 0.22 and 0.20 cm, respectively. The cisterna magna was too small and not enough blood was injected, and the vasospasm severity was 14 and 16 %, respectively, while in other rabbits, the severity was higher than 30 %. In the manual group, no obvious vasospasm was found in three (8 %) rabbits, which indicates the failure of model’s establishment (Table 1).

**Table 1:** Vasospasm severity of basilar artery before and after modeling (number/ %)

Severity of vasospasm	Manual puncture		Ultrasound guided puncture	
	Control group	Model group	Control group	Model group
Severe (≥30 %)	0/0	26/65	0/0	38/95
Moderate (20 ~ 29 %)	0/0	7/18	0/0	0/0
Mild (10 ~ 19 %)	0/0	4/10	0/0	2/5
Small (<10 %)	40/100	3/8	40/100	0/0

## Discussion

There are two basic requirements in the establishment of cerebral vasospasm model: one is subarachnoid hemorrhage, and the other is sufficient stimulation of blood. The methods are varied for different subarachnoid hemorrhage models, they are usually as follows [38]: (1) Let the blood coagulate around the blood vessels by pricking intracranial arteries. (2) After surgical exposure of the experimental arteries, the blood from other parts of the body is injected around the vessels. (3) Autologous blood is injected into cerebral cistern, ventricle or subarachnoid space percutaneously, and the blood coagulates around blood vessels with the flow of cerebrospinal fluid. These three methods have their inherent advantages and disadvantages. The former two methods have many disadvantages such as their associated major trauma and high mortality rates. Double injections of blood into cisterna magna allow high-precision control of injection volume, velocity and time. Due to its low mortality rate, this method has good repeatability, which is suitable for single factor analysis under the stable conditions. Due to the importance of pathogenesis of cerebral vasospasm, double injections of blood into cisterna magna were used to establish the cerebral vasospasm model successfully in rats [39, 40], rabbits [25, 26] and dogs [22].

In the process of establishing cerebral vasospasm model, the death of experimental animals seems inevitable in traditional double injections of blood into cisterna magna. This study showed that even when the blood injected into cisterna magna was replaced with normal saline (manual control group), 15 % of the New Zealand rabbits died during the puncture procedure. The mortality rate was slight higher (23 %) in manual model group. However, no significant difference was found between the manual control and model groups, which indicates that the death of rabbits was caused by the puncture procedure itself, not the blood. The cisterna magna is located behind brain stem. Thus, it is easy to damage the vital center inside brain stem, as the entry depth of needle tip is hard to control in the puncture. The rabbits may die as a result of respiratory arrest. In ultrasound guided puncture procedure, the cisterna magna was found to be too small in two rabbits. Because the depth of cisterna magna was less than the length of inclined plane of needle tip, the brain stem would be damaged in traditional manual model group.

Considering the safety of puncture process, a new technology that can monitor entire puncture process is needed to reduce the mortality rate of rabbits. This study modified puncture technology combined with an interventional ultrasound technique. With ultrasound guidance, the cisterna magna, brain stem and external occipital protuberance were displayed. The brain stem remained undamaged because the depth of needle tip entering into cisterna magna was controlled by the operator. To ensure the safety of puncture process, the best puncture direction was designed according the relationship between atlano-occipital fascia and the deepest part of cisterna magna. As the inclined course in cisterna magna increased the activities of needle tip, the tip would not be able to touch brain stem. Before operation, the angle and the depth of puncture can be detected and calculated by ultrasound. The angle was (56 ± 11) degree, while the depth was (2.38 ± 0.81) cm. These data vary in different rabbits and therefore, a uniform puncture scheme is not sufficient for injection of blood into cisterna magna. With ultrasound guidance, no deaths occurred in 80 cases of New Zealand rabbits and the mortality rate was significantly lower than manual group, which confirmed the safety of this new puncture technology.

The next problem to solve is the validity of model pertaining to whether cerebral vasospasm occurs. Cerebral vasospasm is easier to occur as there are two stimulations in double injections of blood into cisterna magna. In this study, no obvious vasospasm was found in three rabbits in the manual group (8 %), which indicated the failure of model’s establishment. In the ultrasound guided control group, cerebral vasospasm occurred in all experimental animals 5 days after modeling, and the proportion of severe vasospasm was as high as 95 %, which was more than that of manual group (65 %).

Two questions should be considered in ultrasound real-time observation. First, the cisterna magna was too small in 5 % of the rabbits. Insufficient blood was injected to prevent intracranial hypertension. In these rabbits, mild vasospasm was found in MRA images 5 days after modeling. This study suggests that the operator should give up modeling when they find a small cisterna magna. Second, in most of second punctures, adhesion was found near the cisterna magna. It is hard to feel the perforation of dura mater when the needles enter into cisterna magna. If traditional manual puncture were selected, the operator may choose a shallow needle, to prevent damage of brain stem. No blood enters into cisterna magna leading the lack of second stimulation of vessels, reducing the incidence of cerebral vasospasm. This may be the most important reason for the lack of vasospasm in 8 % of rabbits in manual model group.

## Conclusions

In summary, the ultrasound guided double injection of blood into cisterna magna not only has a low animal mortality rate, but it also ensures the occurrence of cerebral vasospasm. This method should be further popularized and applied as a safe and effective rabbit model of cerebral vasospasm.
